# Gasdermin D deficiency aborts myeloid calcium influx to drive granulopoiesis in lupus nephritis

**DOI:** 10.1186/s12964-024-01681-z

**Published:** 2024-06-03

**Authors:** Jiani Shen, Feng Li, Xu Han, Dongying Fu, Yiping Xu, Changjian Zhu, Zhou Liang, Ziwen Tang, Ruilin Zheng, Xinrong Hu, Ruoni Lin, Qiaoqiao Pei, Jing Nie, Ning Luo, Xiaoyan Li, Wei Chen, Haiping Mao, Yi Zhou, Xueqing Yu

**Affiliations:** 1 Department of Nephrology, The First Affiliated Hospital, Sun Yat-sen University, Guangzhou, China; 2 NHC Key Laboratory of Clinical Nephrology (Sun Yat-sen University) and Guangdong Provincial Key Laboratory of Nephrology, Guangzhou, China; 3 Department of Gastroenterology, The First Affiliated Hospital, Sun Yat-Sen University, Guangzhou, China; 4 Department of Nephrology, Guangdong Provincial People’s Hospital and Guangdong Academy of Medical Sciences, Guangzhou, China; 5 Guangdong-Hong Kong Joint Laboratory on Immunological and Genetic Kidney Diseases, Guangdong Provincial People’s Hospital and Guangdong Academy of Medical Sciences, Guangzhou, China

**Keywords:** Gasdermin D, Lupus nephritis, Myeloid cell, Granulopoiesis, Calcium influx

## Abstract

Gasdermin D (GSDMD) is emerging as an important player in autoimmune diseases, but its exact role in lupus nephritis (LN) remains controversial. Here, we identified markedly elevated GSDMD in human and mouse LN kidneys, predominantly in CD11b^+^ myeloid cells. Global or myeloid-conditional deletion of GSDMD was shown to exacerbate systemic autoimmunity and renal injury in lupus mice with both chronic graft-versus-host (cGVH) disease and nephrotoxic serum (NTS) nephritis. Interestingly, RNA sequencing and flow cytometry revealed that myeloid GSDMD deficiency enhanced granulopoiesis at the hematopoietic sites in LN mice, exhibiting remarkable enrichment of neutrophil-related genes, significant increases in total and immature neutrophils as well as granulocyte/macrophage progenitors (GMPs). GSDMD-deficient GMPs and all-trans-retinoic acid (ATRA)-stimulated human promyelocytes NB4 were further demonstrated to possess enhanced clonogenic and differentiation abilities compared with controls. Mechanistically, GSDMD knockdown promoted self-renewal and granulocyte differentiation by restricting calcium influx, contributing to granulopoiesis. Functionally, GSDMD deficiency led to increased pathogenic neutrophil extracellular traps (NETs) in lupus peripheral blood and bone marrow-derived neutrophils. Taken together, our data establish that GSDMD deletion accelerates LN development by promoting granulopoiesis in a calcium influx-regulated manner, unraveling its unrecognized critical role in LN pathogenesis.

**Supplementary Information:**

The online version contains supplementary material available at 10.1186/s12964-024-01681-z.

## Introduction

Systemic lupus erythematosus (SLE) is an autoimmune disease characterized by immune dysregulation and multi-organ involvement [1]. Lupus nephritis (LN) occurs in 35–45% of patients with SLE [2], and is one of the leading causes of mortality and morbidity in SLE patients [3]. The pathogenesis of LN is attributed to autoantibody production, immune complex deposition, and immune cell infiltration in the kidney [4]. Among kidney infiltrating cells, myeloid cells have emerged as important players in the disease, directly participating in key links of immune disorders such as antigen-uptake and presentation, formation of neutrophil extracellular traps (NETs), and cytokine production [5]. The aberrancies of myeloid cells in lupus could be traced back to haematopoietic stem and progenitor cells [6]. Previous researches have depicted strong granulopoiesis signatures in the blood and bone marrow of SLE patients, as a hallmark of active SLE [6–9]. Further studies proved the presence of granulopoiesis in the bone marrow and spleens of New Zealand Black × White (NZB/W) F1 lupus mice, and demonstrated that enhanced granulopoiesis could sustain the inflammatory response and increase the risk for flare in LN [6, 10]. However, the regulatory mechanism of granulopoiesis in lupus remains unknown.

Gasdermin D (GSDMD), the pore-forming effector protein, is involved in diverse inflammatory and autoimmune diseases [11–14]. Upon activation by inflammasome complexes, GSDMD can be cleaved into the N-terminal domain (GSDMD-N), which oligomerizes and forms pores in the plasma membrane [15]. Despite the primary function of GSDMD is considered to be the induction of pyroptosis, increasing evidence suggests that GSDMD executes non-lytic functions in living cells, including the release of cytokines or alarmins and the regulation of ion fluxes [15, 16]. The roles of GSDMD in certain diseases are also diversified and even contradictory within one single disease. This is the case with SLE/LN. Pioneering related work has revealed that global knockout of GSDMD exacerbated autoimmunity and renal inflammation in a TLR7 agonist-induced lupus model without elucidating the mechanism [13]. Paradoxically, recent follow-on studies found that neutrophils-specific GSDMD deficiency or administration of the GSDMD inhibitor disulfiram/Ac-FLTD-CMK alleviated disease severity in pristane-induced lupus model, in which the assembly of GSDMD pores promoted NETs formation [14, 17, 18]. Suspiciously, these studies simultaneously found that GSDMD is expressed not only on neutrophils but also on a variety of other infiltrating immune cells. The greatest existing divergence concerns the opposing phenotypes following GSDMD ablation, which may be partially interpreted by the reported discrepant effects of GSDMD in different cell types. Given that the cellular localization and function of GSDMD in the LN kidney are arguably as yet uncertain, the appropriateness of GSDMD inhibition in LN treatment is unknown.

Here, we demonstrated that GSDMD was up-regulated with disease progression in human and multiple murine LN kidneys, predominantly expressing on myeloid cells without pyroptosis. GSDMD deletion and myeloid-intrinsic GSDMD deficiency in lupus mice significantly exacerbated systemic autoimmune and renal damages, with abnormal granulopoiesis manifested by elevated immature neutrophils and granulocyte/macrophage progenitors (GMPs). Mechanistically, GSDMD knockdown enhanced the self-renewal and differentiation capacity of progenitor cells by inhibiting calcium influx, thereby promoting granulopoiesis to replenish neutrophils pool and exacerbated inflammatory injury in LN. Altogether, our study focuses on neutrophil development and granulopoiesis from a new perspective and reveals a protective effect of myeloid GSDMD on this process and LN, which sheds new light on understanding the role of GSDMD and prompts a rethinking for GSDMD intervention in LN.

## Materials and methods

### Patient samples

All patients fulfilled the American College of Rheumatology classification criteria for SLE [19], and those with biopsy-proven LN according to the International Society of Nephrology/Renal Pathology Society (ISN/RPS) classification [20] were selected as the study population. Patients with infections, tumors, severe underlying disorders or receiving dialysis were excluded. Paraffin-embedded renal specimens of LN patients were collected from diagnostic biopsies and processed for immunohistochemical staining. Normal renal specimens were obtained from para-carcinoma kidney tissues of renal cell carcinoma. Peripheral blood samples of LN patient were collected, and neutrophils were isolated using negative selection via MACSxpress Whole Blood Neutrophil Isolation Kit (Miltenyi Biotec, Cat# 130–104-434) according to the manufacturer’s instructions. The demographic characteristics of LN patients were shown in Tables 1 and 2.

### Animals

Wild-type, GSDMD-deficient and *Gsdmd*^*fl/fl*^ mice were purchased from GemPharmatech Co., Ltd, China. *Gsdmd*^*fl/fl*^ mice were crossed with Lyz2-cre mice (Jackson laboratory, stock# T003822) to generate myelocyte-restricted GSDMD deficiency mice (*Gsdmd*^*△Lyz2*^). The above mice were all on a C57BL/6 background. Female NZB/W F1 mice (stock# 100,008) were obtained from the Jackson Laboratory. All mice were maintained in the specific pathogen-free barrier facility at the Experimental Animal Center at Sun Yat-sen University.

### Animal experiments

To generate cGVH disease mice, donor splenocytes were prepared from B6(C)-*H2-Ab1*^*bm12*^/KhEgJ (bm12) mice (Jackson Laboratory, stock# 001162). 8–12-week-old female C57BL/6 recipient mice were randomized to receive intraperitoneally injections of 1 × 10^8^ donor splenocytes in 200 μl phosphate-buffered saline (PBS) or to receive PBS injections only. All mice were sacrificed 10 weeks post splenocyte grafting, and blood and urine samples were obtained from experimental mice on the day before transplant and biweekly thereafter.

For NTS model induction, 8–12-week-old female C57BL/6 mice were preimmunized with 0.2 mg sheep IgG (GenXion, China) dissolved in complete Freund adjuvant (Sigma-Aldrich, Cat# F5881) via intraperitoneal injection on Day 0. On Day 4, mice received either an intravenous injection of 50 μl Sheep Anti-Rat Glomerular Basement Membrane (GBM) Serum (Probetex, Cat# PTX-001AGBM) or PBS. On Day 11, the mice were sacrificed. Blood and urine were collected at baseline and endpoint of the experiment. Animal experiments were conducted in accordance with the procedure approved by the Institutional Animal Care Committee of Sun Yat-sen University.

### Histology, immunofluorescence and immunohistochemistry

Paraformaldehyde-fixed, paraffin-embedded kidney sections (2 µm) were stained with PAS according to standard laboratory procedures. The indices of renal injuries were scored by a blinded reviewer according to previous methods [21, 22].

For immunofluorescence experiments, both frozen and paraffin sections (5 µm) were blocked with Perm/blocking buffer (0.2% Triton X-100, and 10% donkey serum in PBS). Then, sections were stained alone or in combination with antibodies against IgG (Abcam, Cat# ab150113), C3 (Abcam, Cat# ab11862). MFI was calculated with ImageJ.

Immunohistochemistry for GSDMD was performed on paraffin sections (4 µm). Staining with anti-human GSDMD (Sigma-Aldrich, Cat# HPA044487), anti-human GSDMD-N (Dr. Feng Shao’s lab) or anti-mouse GSDMD (Santa Cruz, Cat# sc393656) was performed according to the instructions of the manufacturers.

### Biochemistry

The concentration of BUN and serum creatinine was measured using automated chemistry analyzer (Roche, COBAS C311) according to the manufacturer's instructions.

### Western blot analysis

Cells or tissues were lysed with protein cracking liquid lysis buffer containing 0.1% protease inhibitor cocktail (Roche, Cat# 5,892,791,001) for 30 min on ice. Lysates were subjected to western blot analysis using the method described previously [23]. The following primary antibodies were used: anti-human GSDMD (Sigma-Aldrich, Cat# HPA044487; RRID: AB_2678957), anti-mouse GSDMD (Abcam, Cat# ab219800; RRID:AB_2888940), anti-human/mouse β-actin (Cell Signaling Technology, Cat# 3700S), anti-GAPDH (Abcam, Cat# ab8245; RRID:AB_2107448).

### Enzyme-linked immunosorbent assay (ELISA)

Serum was collected at the indicated time points to examine anti-dsDNA (Alpha Diagnostic International, Cat# 5120), anti-ssDNA (Alpha Diagnostic International, Cat# 5320), and anti-histone (Alpha Diagnostic International, Cat# 5610) autoantibodies by ELISA according to the manufacturer's instructions. The urine albumin was assessed by the ELISA kit (Clone Cloud-Clone, Cat# CEB028Mu).

### Cell isolation

In mice, tissues were harvested following perfusion with PBS. Kidney tissues were minced and digested in RPIMI-1640 (Gibco) with 2% fetal bovine serum (FBS) (Gibco), 1 mg/ml Type II Collagenase (Gibco) and 0.5 mg/ml Dispase (Gibco) at 37° C with shaking at 200 rpm for 30 min. Following digestion, cell suspensions were filtered with 70 µm cell strainers, centrifuged, and suspended in PBS. Spleens and bone marrow were processed by passing them through 70 µm cell strainers. Red blood cells were lysed with ACK buffer (Thermofisher) and incubated at 4℃ for 5 min. Blood was collected in EDTA-treated tubes and stained with antibodies, and leukocytes were collected and fixed by 1-step fix-and-lyze buffer (Thermofisher, Cat# 00–5333-57).

### Flow cytometry and cell sorting

Cells were gathered and suspended in PBS. Fluorophore-conjugated antibodies were purchased from ebioscience (used at 1:100 dilution): CD45 eFluor™ 450 (clone 30-F11; RRID: AB_1518806), B220 eFluor™ 450 (clone RA3-6B2; RRID: AB_ 2,637,455), CD11c PE-Cyanine7 (clone N418; RRID: AB_ 469,590), F4/80 Super Bright™ 436 (clone BM8; RRID: AB_ 2,723,152), MHC II-A/I-E PerCP-eFluor™ 710 (clone M5/114.15.2; RRID: AB_ 1,834,439), CD317 eFluor™ 450 (clone eBio927; RRID: AB_ 2,043,879), Siglec H FITC (clone eBio440c; RRID: AB_ 837,163), Ly6G FITC (clone 1A8; RRID: AB_ 2,572,532), Ly6C APC (clone HK1.4; RRID: AB_ 1,724,153), CD11b Super Bright™ 600 (clone M1/70; RRID: AB_ 2,637,408), F4/80 PE (clone BM8; RRID: AB_ 465,923), CD4 eFluor™ 506 (clone RM4-5; RRID: AB_ 2,637,459), CD8 FITC (clone 53–6.7; RRID: AB_ 464,915), RORγt APC (clone AFKJS-9; RRID: AB_10609207), Foxp3 PE (clone FJK-16 s; RRID: AB_ 465,936), Gata-3 PE (clone TWAJ; RRID: AB_ 1,963,600), T-bet APC (clone eBio4B10 (4B10); RRID: AB_ 2,744,712), PD-1 PE (clone J43; RRID: AB_ 466,295), CXCR5 APC (clone SPRCL5; RRID: AB_ 2,573,260), B220 eFluor™ 506 (clone RA3-6B2; RRID: AB_ 2,537,455), CD19 PE-Cyanine7 (clone eBio1D3 (1D3); RRID: AB_ 657,663), CD19 FITC (clone eBio1D3 (1D3); RRID: AB_ 657,666), GL7 PerCP-eFluor™ 710 (clone GL-7 (GL7); RRID: AB_2573807), CD95 (APO-1/Fas) APC (clone 15A7; RRID: AB_10717405), CD16/CD32 (clone 93; RRID: AB_1574975), CD11b (clone M1/70; RRID: AB_467107), CD138 PE-Cy7 (clone 281–2; RRID: AB_2562197), Ly-6G/Ly-6C FITC (clone RB6-8C5; RRID: AB_465314), CD44 PE (clone IM7; RRID: AB_465664), CD62L APC (clone MEL-14; RRID: AB_469410), CD34 eFluor™ 450 (clone RAM34; RRID: AB_2043837), CD101 PE (clone Moushi101; RRID: AB_1210728), CD16/CD32 Percp-Cy5.5 (clone 2.4G2 (RUO); RRID: AB_1645259), NK1.1 FITC (clone PK136; RRID: AB_313393), CD90.2 FITC (clone Thy1.2), Sca-1FITC (clone D7; RRID: AB_313343). The fixable viability dye eFluor780 was purchased from Thermo Fisher (used at 1:1000). Cells were dyed at 4 °C, protected from light for 30 min, then centrifuged, washed with PBS and analyzed using Attune NxT (Thermo Fisher Scientific) or Cytek Aurora (Cytek). Data were analyzed using FlowJo software. Lineage (Lin) markers includes CD90.2 (clone Thy1.2), B220 (clone RA3-6B2), Ter119 (TER-119), NK1.1 (clone PK136), Sca-1 (clone D7) and Ly6G (clone 1A8). For sorting, GMPs were purified with FACSAria Fusion (BD) and identified as Lin^−^cKit^+^CD16/32^hi^CD34^+^.

### Quantitative RT-PCR (qRT-PCR)

RNA from neutrophils was extracted with TRIzol (Invitrogen, Cat# 15,596–026). Complementary DNA (cDNA) was synthesized using Hiscrip® II Q RT Supermix (Vazyme), and Quantitative reverse transcription PCR (qRT-PCR) was performed using SYBR Green Master Mix (Roche). Relative expression of target genes was calculated by comparing them to the expression of the housekeeping gene *Gapdh*. The primers sequences that were used for qRT-PCR are the following:

- *Gsdmd*: 5’- ATCCTGGCATTCCGAGTGG -3’(forward);
- 5’-CTCTGGCCCACTGCTTTTCT-3’(reverse),
- *Gapdh*: 5’- ACAACTTTGGTATCGTGGAAGG -3’(forward);
- 5’- GCCATCACGCCACAGTTTC -3′(reverse).

### RNA-seq analysis

CD11b^+^ cells isolated from spleens of GSDMD^*fl/fl*^ cGVH mice or *Gsdmd*^*△Lyz2*^ cGVH mice (*n* = 3 per group) using anti-CD11b-conjugated magnetic beads (Miltenyl Biotec, Cat# 130–049-601) were applied for total RNA extraction with TRIzol (Invitrogen, Cat# 15,596–026) and subjected to RNA-sequencing analysis. RNA quality was assessed on an Agilent 2100 Bioanalyzer (Agilent Technologies, Palo Alto, CA, USA) and checked using RNase free agarose gel electrophoresis. After total RNA was extracted, eukaryotic mRNA was enriched and reverse-transcripted into cDNA. The cDNA libraries were sequenced on the Illumina sequencing platform by Genedenovo Biotechnology Co., Ltd (Guangzhou, China). The raw reads were filtered and mapped to the reference genome using g HISAT2. 2.4. Gene expression levels represented as FPKM (fragment per kilobase of transcript per million mapped reads) value were quantified by the RSEM software. RNA differential expression analysis was performed by DESeq2 software. The genes/transcripts with the parameter of false discovery rate below 0.05 and absolute Log(2) fold change ≥ 1.2 were considered as differentially expressed genes (DEG). DEG were analyzed by the reactome database for pathway enrichment analysis. For hierarchical clustering, euclidean distance and the Ward aggregation criterion were used to plot as a heatmap. Gene ontology (GO) enrichment (GO Biological Process 2021) of DEGs was done using Enrichr.

### Cell culture and transfection

NB4 cells, gifts from Wang Wentao laboratory team (Sun Yat-sen University, Guangzhou, China), were cultured in RPMI-1640 medium (Gibco) with 10% FBS (Gibco) and 1% Penicillin/Streptomycin (Gibco) at 37 °C and 5% CO2. SF Cell Line 4D X Kit L (Lonza, Cat# V4XC-2012) was used according to the manufacturer's protocol. Briefly, NB4 cells (1 × 10^7^) were transfected with 300 nM of siRNA (in 100 μl volume) using CZ-100 program and then rested in complete RPMI 1640 medium (2 × 10^5^ cells/ml) for 12 h, and then treated with RPMI 1640 medium with 1 μM ATRA (Sigma-Aldrich, Cat# R2625) ± 1 μM ionomycin (MCE, Cat# HY-13434) for 24 h. The siRNA was purchased from HanYi Biosciences Inc (Guangzhou, China). The RNA sequences of RNAi oligunucleotides were as follows:

- *Gsdmd*-siRNA-forward: 5’- GUGUCAACCUGUCUAUCAATT -3’;
- *Gsdmd*-siRNA-reversed: 5’- UUGAUAGACAGGUUGACACTT-3’.

### Cytospin and Wright-Giemsa staining

NB4 cells were spun onto glass slides using a cytocentritifugue (StatSpin® CytoFuge 2), dried for 10 min, stained with the wright & giemsa stain solution (Coolaber) according to the manufacturer’s protocol.

### Colony-forming unit‑fibroblasts (CFU‑F) assay

The stem cell potential of the GMPs and NB4 cells was analyzed by the CFU-F assay. Briefly, splenic GMPs (1 × 10^4^ cells/well) or NB4 cells (1 × 10^3^ cells/well) were cultured in 6-well plates using MethoCult M3134 (Stem cell, Cat# 03134) with 10% FBS and 1% Penicillin/Streptomycin, and GMPs were supplemented with SCF (50 ng/ml, Stem cell, Cat# 78,064.1), IL-3 (10 ng/ml, Stem cell, Cat# 78,042.1), GM-CSF (20 ng/ml, PeproTech, Cat# 315–03-20) and G-CSF (20 ng/ml, Stem cell, Cat# 78,014.1). After incubation at 37 °C in a 5% CO_2_ humidified incubator for 7 days, clone cluster were counted with microscope (OLYMPUS), and then cells were collected, washed with PBS, and counted by cell-count boards.

### Calcium measurement

Primary splenic cells or NB4 cells (3 × 10^6^ cells/well) were incubated in PBS (calcium free) with Indo-1, AM (Invitrogen, Cat# I1223) (3 μM) and probenecid (MCE, Cat#HY-B0545) (2.5 μM) at 37°C, protected from light for 15 min. Then, the cells were centrifuged, washed with PBS for two times and analyzed by flow cytometry using Cytek Aurora (Cytek). Stimuli (1μM ATRA or 2 mM calcium) were added as indicated and the samples analyzed continuously.

### NETs formation

Neutrophils from bone marrow were isolated by the gradient density centrifugation described before [24]. Neutrophils (2 × 10^5^ cells/ml) were seeded in complete RPMI 1640 medium in 96-well plates or µ-slide 8 well chamber slide (Ibidi, Cat#80,826) and then stimulated with A23187 (5 uM, MCE, Cat#HY-N6687) or vehicle for 3–4 h under 37℃. CitH3 (Abcam, Cat#ab5103) was used to quantify NETs formation according to manufacturer’s instructions. MPO and cfDNA in the serum were determined by CUSABIO (Thermofisher, Cat#CSB-E08723m) and Picogreen dye (Thermofisher, Cat#P7589) respectively according to manufacturer’s instructions.

### Statistical analysis

All data were expressed as mean ± SEM. Statistical analyses were performed using GraphPad Prism 8. The differences were assessed by Student’s t test, one-way analysis of variance (ANOVA) test, or two-way ANOVA test, wherever as appropriate. For correlation analysis, Pearson correlation test was applied as appropriate. Two-tailed *P* value < 0.05 is considered statistically significant.

## Results

### GSDMD deletion exacerbates lupus-like phenotype in cGVH and NTS mice

**Fig. 1:**
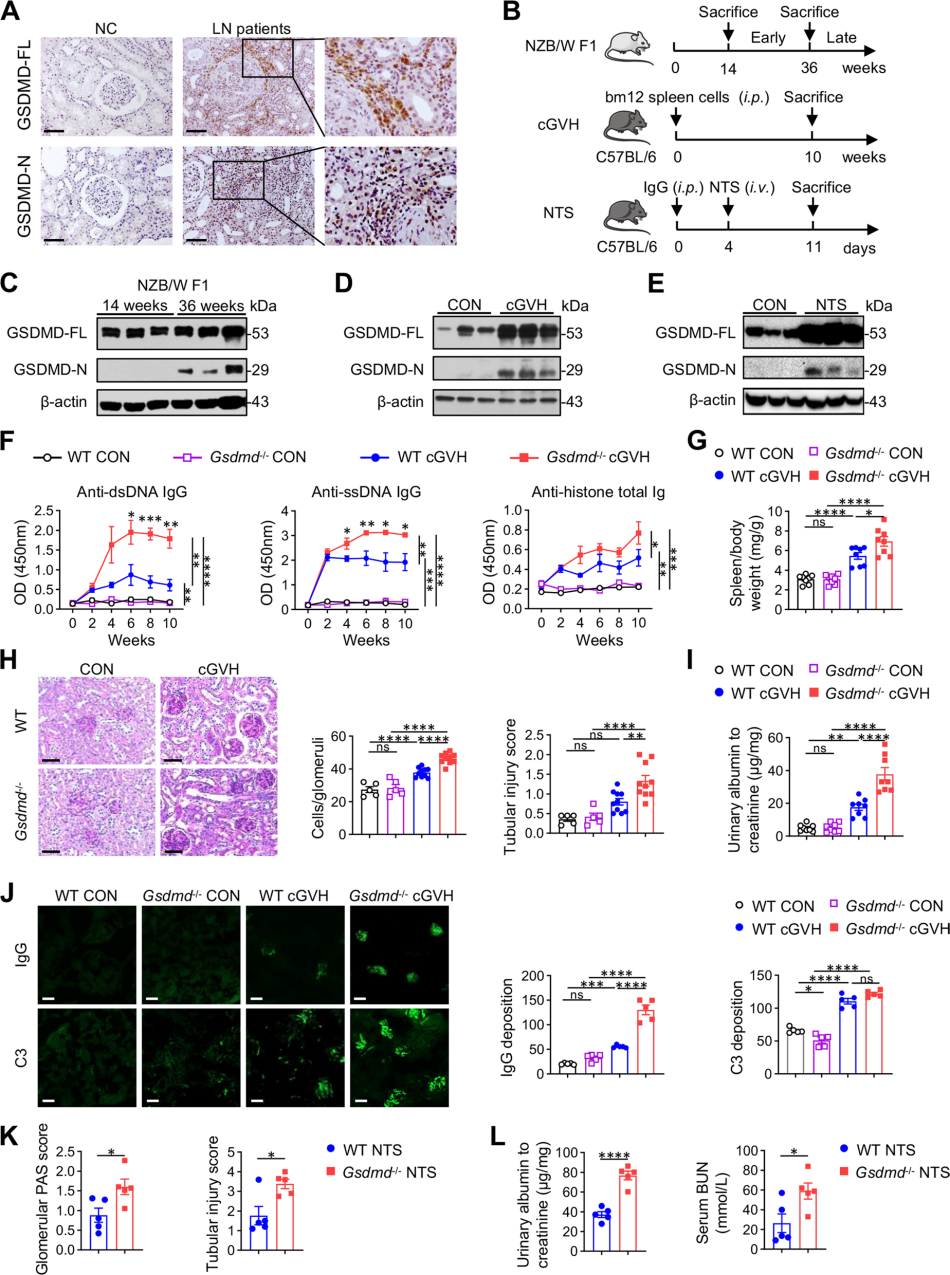
GSDMD deletion exacerbates lupus-like phenotype in cGVH and NTS models. **A** Immunochemical images of GSDMD-FL and GSDMD-N pattern in renal section from normal control (NC) and LN patients. Scale bars: 50 μm. **B** Schematic of murine lupus models. NZB/W F1 mice were sacrificed at 14 weeks and 36 weeks. For cGVH model, C57BL/6 mice received an intraperitoneal injection of 1 × 10^^8^ bm12-derived splenocytes and were sacrificed 10 weeks post-induction. For NTS model, C57BL/6 mice were preimmunized with 0.2 mg sheep IgG intraperitoneally, followed by an intravenous injection of 50 μl sheep NTS on Day 4, and sacrificed on Day 11. **C-E** Immunoblotting of GSDMD in renal protein extracts of NZB/W F1 mice, cGVH or NTS mice with control (CON) mice. β-actin is loading control. **F** ELISA determining the level of autoantibodies (anti-dsDNA, anti-ssDNA and anti-histone) in serum of cGVH mice (*n* = 4 or 5 per group). **G** Spleen to body weight ratio in cGVH mice (*n* = 8 per group). **H** Representative PAS staining of kidney sections with quantitative analysis of glomerular cellularity and tubular injury scores in cGVH mice (*n* = 5 or 10 per group). Scale bars: 50 μm. **I** Urine albumin to creatinine ratio in cGVH mice (*n* = 8 per group). **J** Representative images and quantified immunofluorescence density of IgG and C3 deposition in the kidneys from cGVH mice (*n* = 5 per group). Scale bars: 50 μm. **K** Quantitative analysis of glomerular PAS score and tubular injury scores in kidney sections from NTS mice (n = 5 per group). **L** Urine albumin to creatinine ratio and BUN in NTS mice (n = 5 per group). Data are shown as mean ± SEM. Two-way ANOVA test, ANOVA or Student’s t test was used for statistical analysis. ****P < 0.0001; ***P < 0.001; **P < 0.01; *P < 0.05; ns, not significant

To characterize the expression of GSDMD in LN kidney, we detected GSDMD in human kidney sections by immunohistochemistry, and found that both GSDMD full-length (GSDMD-FL) and its active fragment GSDMD-N were significantly up-regulated in LN patients compared with normal kidneys, especially in infiltrating leukocytes that gathered around the glomeruli and tubulointerstitium (Fig. 1A, Fig. 1—figure supplement 1A). Next, we validated the same GSDMD expression pattern in three widely used lupus models, including spontaneous lupus mouse NZB/W F1, bm12 splenocyte-induced cGVH model [25, 26] and NTS nephritis [27, 28] (Fig. 1B). Accordantly, GSDMD was also elevated and activated in the kidneys of these LN models compared to their normal controls (Fig. 1C-E, Fig. 1—figure supplement 1B-G).

To assess the role of GSDMD in the development of LN, we first established two inducible LN models using GSDMD-deficient mice to define the phenotype. Following cGVH induction, GSDMD deletion exhibited more severe lupus-like systemic autoimmune disorders than wild-type controls, with markedly increased serum autoantibodies against dsDNA, ssDNA and histones (Fig. 1F), as well as enhanced splenomegaly (Fig. 1G). These differences were absent between the non-immunized groups (Fig. 1F-G). Regarding kidney involvement, GSDMD knockout showed more pronounced glomerular hypercellularity, tubular injury score (Fig. 1H), urine albumin-to-creatinine ratio (ACR) (Fig. 1I), and intraglomerular IgG deposition (Fig. 1J), indicating that cGVH mice developed a more severe lupus-like nephritis phenotype in the absence of GSDMD. Meanwhile, we applied NTS model to focus on immune complexes-mediated glomerulonephritis by nephrotoxic serum immunization (Fig. 1B). Compared to wild-type, GSDMD-deficient NTS kidneys suffered greater damage, as evidenced by greater staining for Periodic acid-Schiff (PAS)-positive materials, doubled tubular injuries scores in kidney sections (Fig. 1K), and ACR and serum blood urea nitrogen (BUN) levels (Fig. 1L). Collectively, the consistent performance on both models suggests that GSDMD deficiency produces higher autoantibodies and exacerbates renal injury in LN.

## Myeloid GSDMD deficiency is sufficient to exacerbate autoimmunity and renal injury in lupus mice

**Fig. 2:**
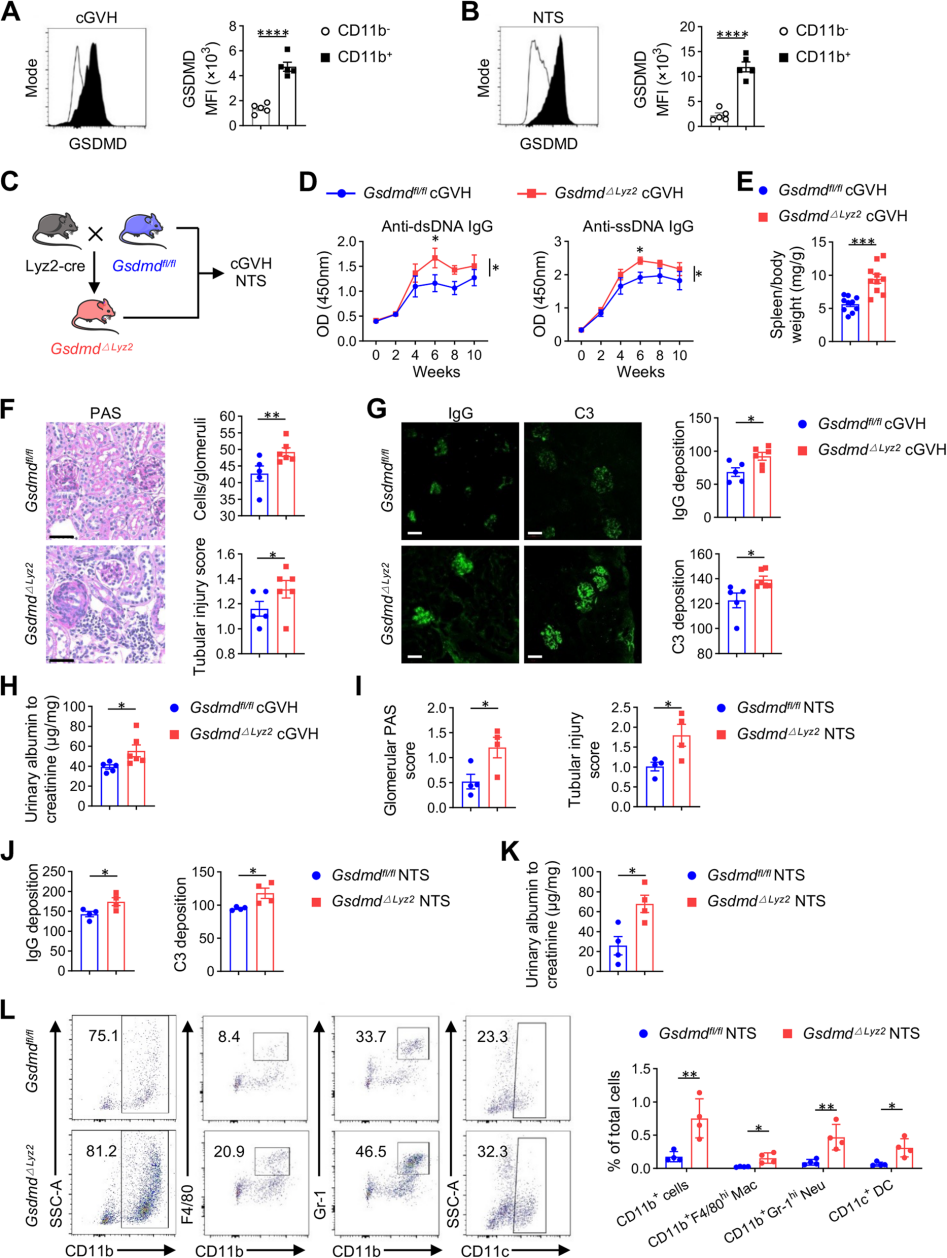
Myeloid GSDMD deficiency is sufficient to exacerbate autoimmunity and renal injury in lupus mice. **A-B** The MFI of GSDMD expression in CD11b^+^ cells and CD11b^−^ cells from cGVH and NTS models (n = 5 per group). **C** Experimental outline of the generation of myeloid-specific GSDMD-deficient mice (*Gsdmd*^*△Lyz2*^) and LN models construction in *Gsdmd*^*△Lyz2*^ and its littermate controls (*Gsdmd*^*fl/fl*^). **D** ELISA determining the level of autoantibodies (anti-dsDNA and anti-ssDNA) in the serum of cGVH mice (*n* = 6 or 7 per group). **E** Spleen to body weight ratio in *c*GVH mice (*n* = 10 per group). **F** Representative PAS staining and quantitative analysis of glomerular cellularity and tubular injury scores of kidney sections from cGVH mice. Scale bar: 50 μm. (*n* = 5 or 6 per group). **G** Immune complex deposition (IgG and C3) immunofluorescence of kidney sections from different groups of cGVH mice. Scale bar: 50 μm. Quantitative analysis of IgG and C3 MFI were analyzed (*n* = 5 or 6 per group). **H** Urine albumin-to-creatinine ratio in different groups of cGVH mice (*n* = 5 or 6 per group). **I** Quantitative analysis of glomerular PAS score and tubular injury scores of kidney sections from NTS mice (n = 4 per group)**. J** Quantitative analysis of IgG and C3 MFI of kidney sections from NTS mice (*n* = 4 per group). **K** Urine albumin-to-creatinine ratio in NTS mice (*n* = 4 per group). **L** Representative image of flow cytometric analysis and quantitative analysis of the percentages of CD11b^+^ cells, CD11b^+^F4/80^hi^ macrophages (Mac), CD11b^+^Gr-1^hi^ neutrophils (Neu) and CD11c^+^ dendritic cells (DC) among total cells in the kidneys of NTS mice (n = 4 per group). Data are shown as mean ± SEM. One-way ANOVA test, two-way ANOVA or Student’s t test was used for statistical analysis. ****P < 0.0001; ***P < 0.001; **P < 0.01; *P < 0.05

To gain insight into the cellular localization of GSDMD during LN development, we examined the expression of GSDMD on CD11b^+^ cells or CD11b^−^ cells in the kidneys of cGVH and NTS models, in which flow analysis revealed that the expression intensity of GSDMD was 2 ~ 5 fold higher in CD11b^+^ cells than that in CD11b^−^ (Fig. 2A-B, Fig. 2—figure supplement 1A). Since activated GSDMD has a non-negligible role in mediating myeloid pyroptosis [29], we further analyzed the occurrence of pyroptosis under lupus induction. Unexpectedly, myeloid pyroptosis was not increased with elevated and activated GSDMD during LN, represented as the comparable percentages of caspase-1/11^+^PI^+^ cells or caspase-1/11^+^Hoechst^+^ cells gated on CD11b^+^ cells in the kidneys of lupus mice and their controls (Fig. 2—figure supplement 1B-C). Therefore, GSDMD deficiency participates in LN not by causing myeloid pyroptosis but through other mechanisms.

Given that GSDMD predominates in infiltrating CD11b^+^ myeloid cells in LN kidneys (Fig. 2A, B), we next generated Lyz2-cre × *Gsdmd*^*fl/fl*^ (*Gsdmd*^*△Lyz2*^) mice with myelocyte-restricted GSDMD deficiency to investigate the role of myeloid-intrinsic GSDMD (Fig. 2C). Consistent with the global deletion of GSDMD, we found that myeloid-intrinsic GSDMD-deficient mice developed elevated levels of serum autoantibodies against dsDNA and ssDNA (Fig. 2D), and a significantly more severe splenomegaly **(**Fig. 2E) compared with wild-type littermates. In addition, *Gsdmd*^*△Lyz2*^ mice also possessed significantly increased numbers of Tfhs and plasma cells in spleens compared to disease controls (Figure. 2—figure supplement 1D-F), suggesting that the absence of myeloid-intrinsic GSDMD enhanced systemic autoimmune responses in LN.

Moreover, *Gsdmd*^*△Lyz2*^ cGVH mice showed exacerbated kidney damage compared with *Gsdmd*^*fl/fl*^ controls, including higher glomerular cellularity and tubular injury score (Fig. 2F), as well as IgG and C3 deposition (Fig. 2G) and elevated ACR (Fig. 2H). Likewise, in NTS mice, the pathological scores of *Gsdmd*^*△Lyz2*^ were twice that of control mice (Fig. 2I), and *Gsdmd*^*△Lyz2*^ mice had more serious immune complex deposition (Fig. 2J) and urine albumin excretion (Fig. 2K). Increased inflammatory infiltration of immune cells, especially CD11b^+^ myeloid cells, including CD11b^+^F4/80^+^ macrophages, CD11b^+^Gr-1^+^ neutrophils and CD11c^+^ dendritic cells (DC), was also observed in both the *Gsdmd*^*△Lyz2*^ NTS and cGVH kidneys (Fig. 2L, Fig. 2—figure supplement 2G). Taken together, myeloid-intrinsic GSDMD deficiency is sufficient to recapitulate the effects of global GSDMD knockout and promote renal injury and inflammation during LN.

Notably, when screening for changes in immune cell subsets in the spleens of lupus mice, we found that loss of GSDMD did not make a difference in total B cells, T cells differentiation into T helper (Th1, Th2, and Th17) or Treg cells, as well as the type I interferon-producing plasmacytoid dendritic cells (pDCs) (data not shown), indicating that GSDMD likely does not act through these lymphocytes and pDCs.

## GSDMD-deficient myeloid cells from lupus spleen exhibit immature neutrophil expansion and differentiation

**Fig. 3:**
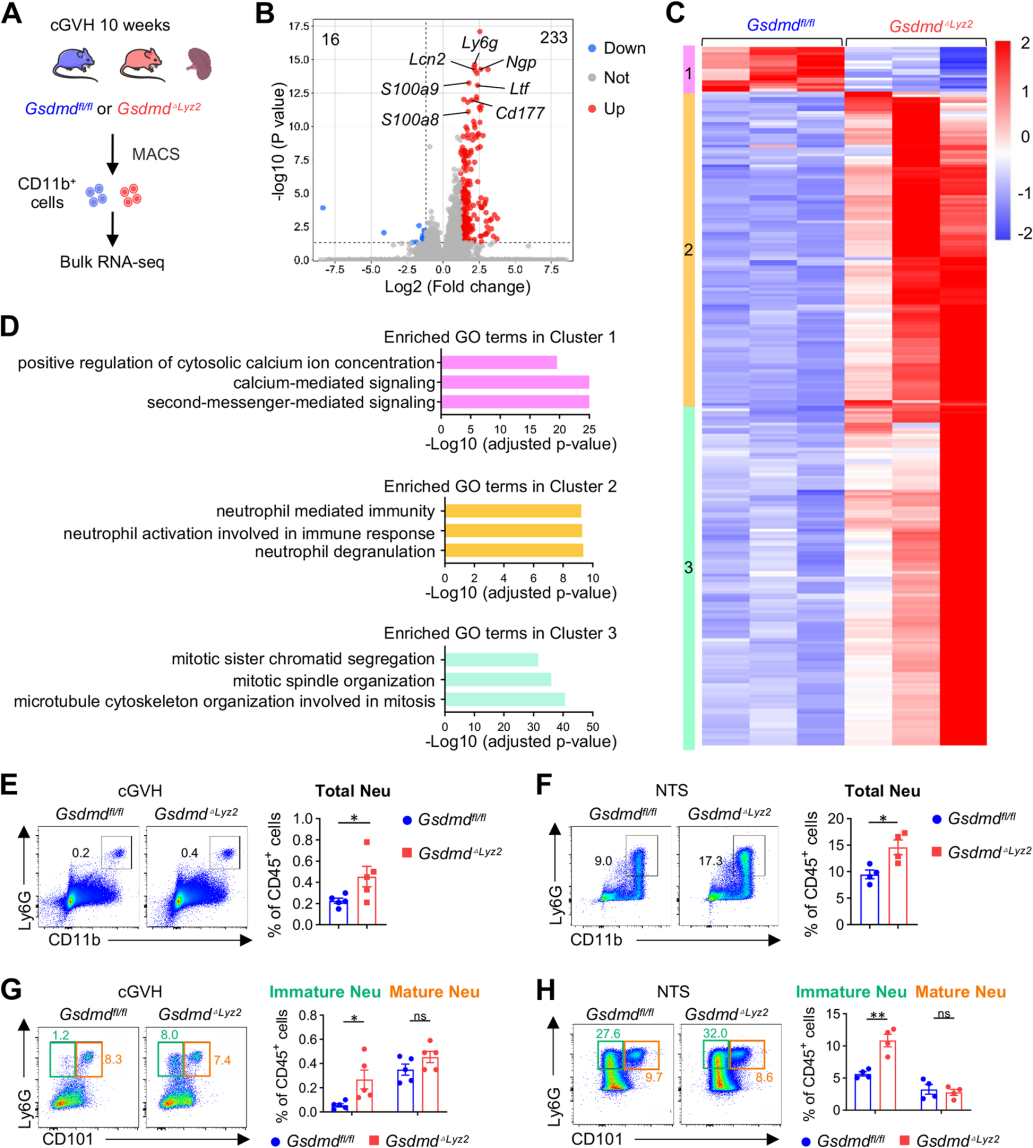
GSDMD-deficient myeloid cells from lupus spleen exhibit immature neutrophil expansion and differentiation. **A** Experimental design for RNA-sequencing. *Gsdmd*^*fl/fl*^ and *Gsdmd*^*△Lyz2*^ mice were induced cGVH model. After 10 weeks, mice were sacrificed, and then splenic CD11b^+^ cells were isolated using magnetic-activated cell sorting (MACS) and performed bulk RNA-seq (*n* = 3 per group). **B** Volcano plot for DEG of splenic CD11b^+^ cells from *Gsdmd*^*fl/fl*^ and *Gsdmd*^*△Lyz2*^ mice (*n* = 3 per group). **C** Heatmap of DEG from bulk RNA-seq transcripts of sorted CD11b^+^ cells above. Data are represented as a Z score, from low (blue) to high (red) expression. Gene clusters (1 to 3) were defined according to hierarchical clustering. **D** Bar plots of top GO terms from DEGs exported for gene ontology (GO) enrichment analysis showing the top GO terms. **E** Flow cytometric analysis of total CD11b^+^Ly6G^hi^ neutrophils in the spleens of cGVH induced in *Gsdmd*^*fl/fl*^ and *Gsdmd*^*△Lyz2*^ mice (*n* = 4 or 5 per group). **F** Flow cytometric analysis of total CD11b^+^Ly6G^hi^ neutrophils in the spleens of NTS model induced in *Gsdmd*^*fl/fl*^ and *Gsdmd*^*△Lyz2*^ mice (*n* = 4 or 5 per group). **G** Flow cytometric analysis of Ly6G^+^CD101^−^ immature and Ly6G^+^CD101^+^ mature neutrophils in the spleens of cGVH (*n* = 5 per group). **H** Flow cytometric analysis of Ly6G^+^CD101^−^ immature and Ly6G^+^CD101^+^ mature neutrophils in the spleens of NTS model induced in *Gsdmd*^*fl/fl*^ and *Gsdmd*^*△Lyz2*^ mice (*n* = 4 per group).. Data are shown as mean ± SEM. Student’s t test was used for statistical analysis. **P < 0.01; *P < 0.05; ns, not significant

To further explore how GSDMD modulates myeloid cells to protect against lupus, we performed whole-transcriptome sequencing (RNA-seq) analysis using CD11b^+^ cells sorted from the spleens of *Gsdmd*^*fl/fl*^ and *Gsdmd*^*△Lyz2*^ cGVH mice, respectively (Fig. 3A). Taking the Log(2) fold change > 1.2 as the criteria, the volcano plots revealed a total of 233 genes up-regulated and 16 genes down-regulated in CD11b^+^ cells from *Gsdmd*^*△Lyz2*^ diseased mice, among which neutrophil markers were significantly increased, including *Ly6g*, *Lcn2*, *Ngp*, *S100a8*, *S100a9*, *Ltf* and *Cd177* (Fig. 3B). We next plotted all differentially expressed genes in a heat map and identified three distinct clusters (Fig. 3C). GO enrichment analysis highlighted the possibility of GSDMD pore-mediated calcium influx, demonstrated by notable down-regulation of a cluster of genes involved in cytosolic calcium concentration and concomitant calcium-mediated signaling in *Gsdmd*-deficient CD11b^+^ cells (Fig. 3D). Intriguingly, on the other hand, compared with wild-type control cells, *Gsdmd*-deficient CD11b^+^ cells displayed two upregulated gene clusters related to neutrophil function and mitotic cell division (Fig. 3D). These neutrophil expansion, strongly suggested by bioinformatic analyzes, was confirmed when we further examined splenic CD11b^+^Ly6G^+^ neutrophils in the *Gsdmd*^*△Lyz2*^ cGVH (Fig. 3E) and NTS models (Fig. 3F). Following these hints, we turned to investigate neutrophil subpopulations in CD11b^+^ cells of disease-challenged *Gsdmd*^*fl/fl*^ and *Gsdmd*^*△Lyz2*^ mice to explore the reasons behind neutrophil expansion. Interestingly, we observed a nearly 1- or 2-fold increase of immature CD11b^+^Ly6G^+^CD101^−^ neutrophils in the spleen of *Gsdmd*^*△Lyz*^ mice after cGVH or NTS induction, whereas mature CD11b^+^Ly6G^+^CD101^+^ neutrophils did not undergo obvious changes (Fig. 3G-H, Fig. 3—figure supplement 1). Combined with growing population of neutrophils, the unbalanced percentages of immature and mature neutrophils inspire us on the possibility that GSDMD regulates granulopoiesis, which is induced during inflammation and generally characterized by preferential neutrophil differentiation in bone marrow and extramedullary organ such as spleen [30].

### Myeloid GSDMD deficiency promotes systemic granulopoiesis and NETs formation in lupus

**Fig. 4:**
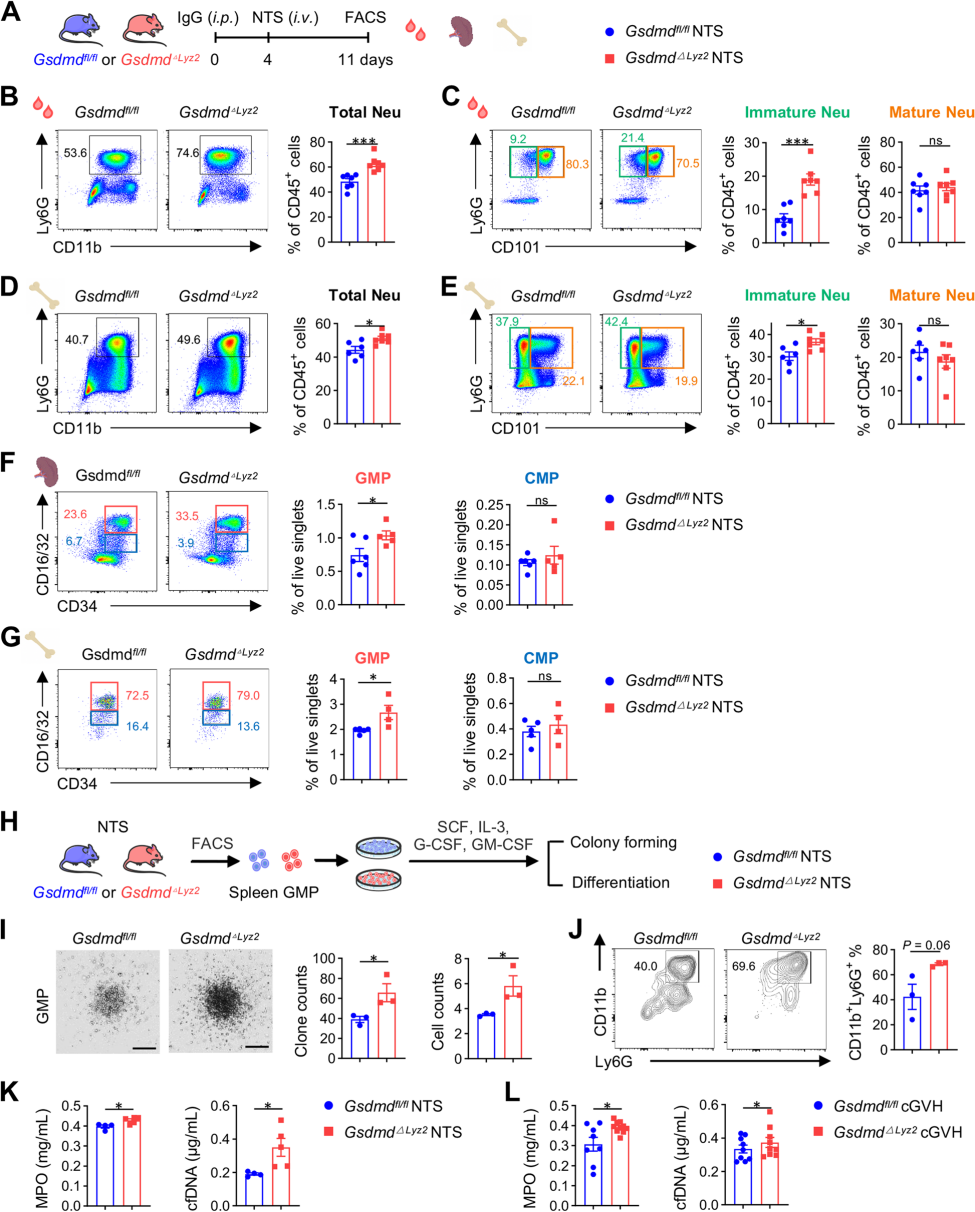
Myeloid GSDMD deficiency promotes granulopoiesis and NETs formation in lupus. **A** Experimental scheme. NTS model was induced in *Gsdmd*^*fl/fl*^ and *Gsdmd*^*△Lyz2*^ mice, and then blood, spleens and bone marrow were collected for flow cytometry. **B** Representative flow cytometric plots and quantification of total CD11b^+^Ly6G^hi^ neutrophils in the blood of NTS mice (*n* = 7 per group). **C** Representative flow cytometric plots and quantification of Ly6G^+^CD101^−^ immature and Ly6G^+^CD101^+^ mature neutrophils in the blood of NTS mice (*n* = 7 per group). **D-E** Representative flow cytometric plots and quantification of total CD11b^+^Ly6G^hi^ neutrophils, Ly6G^+^CD101^−^ immature and Ly6G^+^CD101^+^ mature neutrophils in the bone marrow of NTS mice (*n* = 7 per group). **F** Representative flow cytometric plots and quantification of GMPs and CMPs in the spleens of NTS mice (*n* = 4–6 per group). **G** Representative flow cytometric plots and quantification of bone marrow of NTS mice (*n* = 4–6 per group). **H** Experimental scheme. Splenic GMPs were sorted from *Gsdmd*^*fl/fl*^ and *Gsdmd*^*△Lyz2*^ NTS mice by fluorescence-activated cell sorting (FACS), and stimulated with SCF, IL-3, G-CSF and GM-CSF. **I** Representative images and quantification of colony forming of splenic GMPs (*n* = 3 per group). Scale bars: 200 μm. **J** Representative flow cytometric plots and quantification of CD11b^+^Ly6G^+^ neutrophils (*n* = 3 per group). **K** ELISA determining MPO and cfDNA levels representing NETs release in serum from NTS mice (*n* = 4–5 per group). **L** ELISA determining MPO and cfDNA levels representing NETs release in serum from cGVH mice (*n* = 8–10 per group). Data are shown as mean ± SEM. Student’s t test was used for statistical analysis. ***P < 0.01; *P < 0.05; ns, not significant

To demonstrate that granulopoiesis does occur, we analyzed immature/mature neutrophils and neutrophil progenitors in circulation and hematopoietic organs. In the NTS model (Fig. 4A), as expected, we observed a significant increase in CD11b^+^Ly6G^+^ total neutrophils in blood and bone marrow of *Gsdmd*^*△Lyz2*^ mice compared to *Gsdmd*^*fl/fl*^ control, which was mainly attributed to the increased amount of immature CD11b^+^Ly6G^+^CD101^−^ neutrophils (Fig. 4B-E). Next, we focused on neutrophil progenitors, including GMPs and common myeloid progenitors (CMPs), in the spleen and bone marrow of the hematopoietic sites of *Gsdmd*^*△Lyz2*^ and *Gsdmd*^*fl/fl*^ mice. GMPs were found to be increased in the spleen and bone marrow of *Gsdmd*^*△Lyz2*^ lupus mice (Fig. 4F, G, Figure 4—figure supplement 1). In addition to the amount of GMPs, we further examined their self-renewal ability, another potent evidence for granulopoiesis. By sorting splenic GMPs from NTS mice and culturing them ex vivo for 7 days (Fig. 4H), we found increased clonogenicity of GSDMD-deficient GMPs compared with WT GMPs (Fig. 4I), suggesting that GSDMD-deficiency increases their self-renewal capability. Also, a trend towards a higher proportion of CD11b^+^Ly6G^+^ neutrophils was observed in the same ex vivo system (Fig. 4J). These findings reaffirm our hypothesis that GSDMD deficiency promotes granulopoiesis and neutrophil output during lupus progression.

With the strengthen of granulopoiesis following GSDMD deficiency in myeloid cells, we demonstrated that NETs components, including neutrophil enzymes myeloperoxidase (MPO) and cell-free DNA (cfDNA) [31], were increased in serum from NTS and cGVH mice (Fig. 4K, L). This result suggests that GSDMD deficiency-induced granulopoiesis contributes to the production of NETs in lupus environment.

### GSDMD deficiency promotes granulopoiesis by reducing calcium influx in myeloid progenitors

**Fig. 5:**
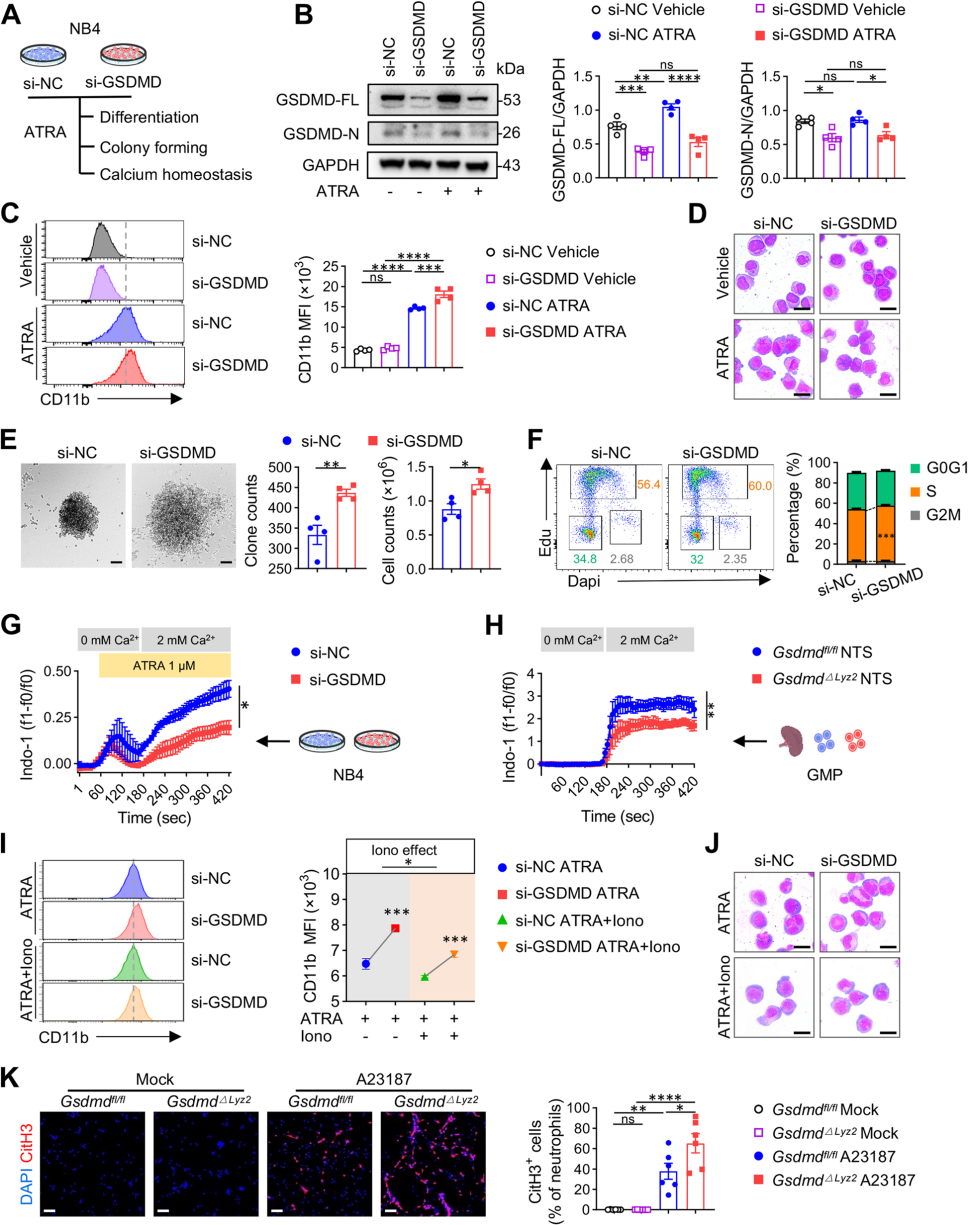
GSDMD deficiency promotes granulopoiesis by reducing calcium influx in myeloid progenitors. **A** Experimental scheme. NB4 cells transfected with si-NC and si-GSDMD were stimulated with ATRA (1μM), and then performed a series of analysis. **B** Immunoblotting of GSDMD in protein extracts of NB4 cells (*n* = 4 per group). GAPDH is loading control. **C** Representative flow cytometric plots and quantification CD11b MFI in different groups of NB4 cells (*n* = 4 per group). **D** Representative micrographs of wright & giemsa staining of NB4 cells. Scale bars: 10 μm. **E** Representative pictures and quantification of colonies of NB4 cells (*n* = 4 per group). Scale bars: 100 μm. (**F**) Cell cycle analysis of NB4 cells (*n* = 3 per group). **G-H** Flow analysis of the intracellular calcium levels using Indo-1, AM (3 μM) in NB4 cells (*n* = 3 per group) or GMPs isolated from the spleens of *Gsdmd*^*fl/fl*^ and *Gsdmd*^*△Lyz2*^ NTS mice (*n* = 5 per group). 1 μM ATRA and 2 mM calcium was added to the cells at the indicated time. **I** Representative plots and quantification of CD11b MFI in ATRA-stimulated NB4 cells treated with or without ionomycin (Iono) (1μM) (*n* = 4 per group). **J** Representative micrographs of wright & giemsa staining of NB4 cells. Scale bars: 10 μm. **K** Representative images and quantified immunofluorescence of NETs release from bone marrow-derived neutrophils isolated from *Gsdmd*^*fl/fl*^ and *Gsdmd*^*△Lyz2*^ NTS mice after 4h treatment with A23187 (5 uM) or vehicle. NETs release was calculated as the ratio of CitH3 (red area) in total cells (DAPI, blue area) per 100 neutrophils using ImageJ (*n* = 6 per group). Scale bars: 50 μm. Data are shown as mean ± SEM. One-way ANOVA, two-way ANOVA test or Student’s t test was used for statistical analysis. ****P < 0.0001; ***P < 0.001; **P < 0.01; *P < 0.05; ns, not significant

Now that GSDMD is involved in the regulation of granulopoiesis, we further investigated whether GSDMD directly affected the differentiation of myeloid progenitors. To this end, we utilized all-trans-retinoic acid (ATRA) treatment of NB4 cells, an acute promyeloid cell line, to induce their differentiation [32] as an in vitro model (Fig. 5A). The expression of GSDMD was increased in NB4 cells after ATRA treatment (Fig. 5B), which was consistent with the elevation of GSDMD we observed in LN. We further depleted GSDMD using siRNA to explore GSDMD’s role in NB4 cell differentiation (Fig. 5B). As shown in Fig. 5C, ATRA induced an increase in the mean fluorescence intensity (MFI) of CD11b in NB4 [33], whereas this response was enhanced by GSDMD deletion, suggesting that knockdown of GSDMD encouraged ATRA-induced differentiation of myeloid progenitors. This result was further verified by the morphological maturity of NB4 cells detected by Giemsa staining (Fig. 5D). Besides, GSDMD knockdown promoted self-renewal ability in NB4 cells, demonstrated by increased colony numbers and cell counts (Fig. 5E). Along with the clonal expansion, we also observed that GSDMD knockdown caused an increased percentage of NB4 cells in the S phase of cell cycle (Fig. 5F), indicating a higher proliferation proportion [34]. Next, we wondered what intracellular mechanisms mediated these changes. Considering the significant enriched calcium-mediated pathways in myeloid cells after GSDMD knockout, and the important role of calcium in cell differentiation [35–38], we thus assumed GSDMD deficiency-induced differentiation was calcium-related. Measuring calcium influx by flow cytometry, we observed that GSDMD deficiency attenuated calcium influx in ATRA-treated NB4 (Fig. 5G), a phenomenon that was also verified in primary GMPs isolated from NTS spleens (Fig. 5H). These data indicated that the increase of cell differentiation was accompanied by a reduction of intracellular calcium concentration in neutrophil precursors. To disentangle the relationship between these changes, we used low-dose ionomycin (a calcium ionophore which promotes calcium influx by stimulating store-regulated calcium entry (SOCE)) [39] concurrently with ATRA treatment to increase cytosolic calcium in NB4 cells. We found that the pro-differential effect of GSDMD deficiency could be compromised by ionomycin, as displayed by the reduced levels of CD11b MFI and morphologically differentiated cells in ionomycin-treated NB4 cells (Fig. 5I-J). Interestingly, the gaps in these metrics between the si-NC and si-GSDMD was narrowed compared to ATRA alone, supporting that GSDMD affects granulopoiesis through mediating calcium entry. Together, these findings indicated that knockdown of GSDMD could drive granulopoiesis by reducing intracellular calcium concentration.

We further verified the impact of GSDMD deficiency on NETs production in in vitro experiments. NETs were first measured in neutrophils isolated from bone marrow of *Gsdmd*^*△Lyz*^ and *Gsdmd*^*fl/fl*^ NTS mice using citrullinated histone H3 (CitH3), a well-defined marker of NETs release [40]. *Gsdmd*^*△Lyz*^ neutrophils displayed stronger ability to form NETs than the controls when stimulated by calcium ionophore A23187 [41] (Fig. 5K), in accord with in vivo results (Fig. 4J-K) and further proving the progressive NETs formation in GSDMD-deficient LN mice.

### GSDMD expression on neutrophils in LN patients is inversely correlated with disease activity and proteinuria

**Fig. 6:**
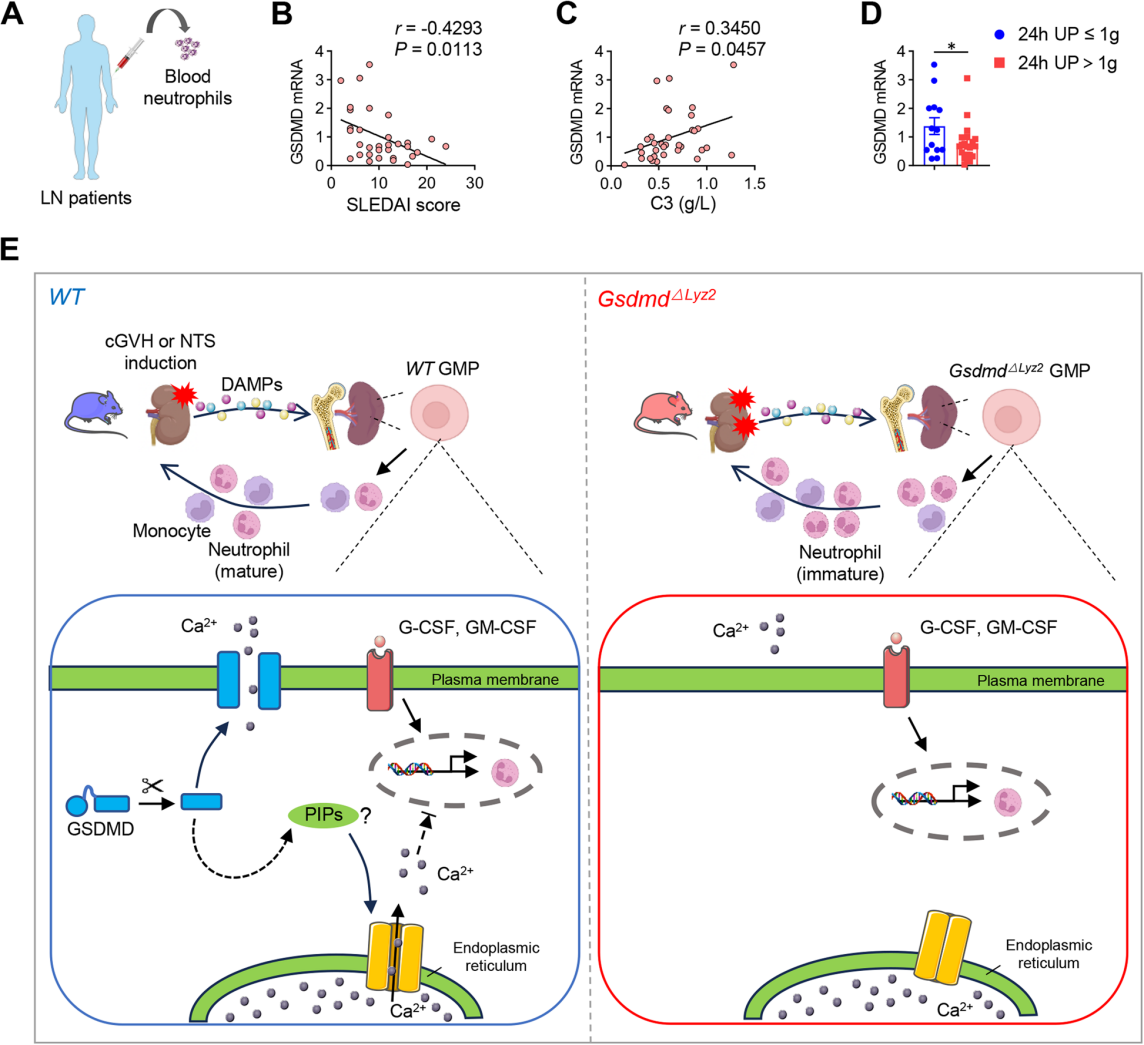
GSDMD expression on neutrophils in LN patients is inversely correlated with disease activity and proteinuria. **A** Neutrophils were isolated from the blood of LN patients to perform RT-PCR. **B** GSDMD expression negatively correlated with SLEDAI score in LN patients (*n* = 32). **C** GSDMD expression positively correlated with serum C3 level (g/L) in LN patients (*n* = 32). **D** GSDMD expression was compared in LN patients with higher 24h urine protein (24h UP) (> 1g) and lower 24h UP (≤ 1g) (*n* = 12 or 20 per group). Data are shown as mean ± SEM. Student’s t test or Pearson correlation test was performed.*P < 0.05. **E** Model for GSDMD function in granulopoiesis during LN. Following the induction of cGVH or NTS, DAMPs are released from the injured kidney, which activate granulopoiesis in the spleen and bone marrow. GSDMD is upregulated and activated in GMPs, potentially facilitating calcium entry into the cytoplasm. This occurs via two possible mechanisms: calcium influx through GSDMD-N pores in the plasma membrane or through Store-Operated Calcium Entry (SOCE) regulated by interactions between GSDMD and phosphoinositides (PIPs). GSDMD deficiency in myeloid cells restricts intracellular calcium concentrations, promotes granulocytic differentiation of GMPs, and subsequently enhances granulopoiesis. This leads to an uncontrolled expansion in pathogenic neutrophils, thereby exacerbating renal impairment in lupus nephritis

Since our results indicated that GSDMD was a negative regulator of granulopoiesis, we then collected neutrophils from LN patients to investigate the relationship of GSDMD level with LN disease severity (Fig. 6A). GSDMD mRNA levels in neutrophils of LN patients were significantly negatively correlated with disease activity as reflected by Systemic Lupus Erythematosus Disease Activity Index (SLEDAI) score (Fig. 6B). Individuals with lower levels of GSDMD mRNA were also associated with lower C3 (Fig. 6C) and heavier proteinuria among LN patients (Fig. 6D). Together, these results suggest that neutrophil GSDMD is negatively correlated with human LN pathogenesis, which may be attributed to the alteration of granulopoiesis.

## Discussion

The role of GSDMD in murine lupus models remains highly controversial. A recent study using global- and neutrophils-conditional GSDMD knockout mice showed that GSDMD aggravated SLE development in a pristane-induced model by mediating NETs formations in neutrophils [14]. However, an under-considered aspect of this study is that besides neutrophils, there are also some other myeloid cells including precursors highly expressing GSDMD, which may present more intricate effects under GSDMD’s regulation. Adding to the confusion, GSDMD-global knockout has previously been shown to increase myeloid cell expansion and accelerate lupus phenotypes in TLR7 agonist- or pristane-induced lupus models [13], which actually coincide with our phenotypic conclusions. The opposite results indicate that GSDMD has extremely complex functions and unknown effects worth exploring. Our study employed cGVH and NTS models, which presented a more severe immune complex-mediated glomerulonephritis phenotype, providing a better simulation of lupus patients with active nephritis [42–44]. In these two models, by utilizing global- and myeloid-conditioned GSDMD-knockout mice, we proved the protective effect of GSDMD in the pathogenesis of LN and proposed that GSDMD was a key mediator of neutrophil homeostasis under lupus conditions, with the potential to stimulate granulopoiesis. Our findings provide new evidence and insights into a comprehensive understanding of the paradoxical functions of GSDMD in lupus, which is indeed important for the rationale of implementing GSDMD interventions in LN.

Granulopoiesis is important for neutrophil homeostasis in a physiological state, while abnormal granulopoiesis can result in persistent inflammation responses [45]. In lupus, the presence of granulopoiesis and its close association with disease severity have been proven by numerous studies [6–10], and growing immature granulocytes also possess the functions of the mature one in aggravating the disease [34, 41, 46]. Yet the mechanism of granulopoiesis in SLE/LN remains scarce, which is fundamental for understanding lupus granulopoiesis and intervening in granulocyte-related inflammation. Our study discovered that GSDMD is a key regulator of granulopoiesis by combining unbiased RNA-sequencing data and a series of validation experiments, which may fill the gap in the regulation of granulopoiesis in lupus. In fact, a relationship between GSDMD and granulopoiesis has been reported in myocardial infarction (MI), albeit in the opposite direction, with GSDMD promoting granulopoiesis through the NLRP3-GSDMD-IL-1β signaling pathway [47]. Such opposing observations with us might be explained by the different immunopathogenesis between diseases, as NLRP3-GSDMD-IL-1β axis exerts definite pro-inflammatory effects in MI but not in lupus-like autoimmunity [13, 47, 48]. Besides, one of the interesting findings in our study is the prominent extramedullary granulopoiesis caused by GSDMD deficiency. Although bone marrow is the main hematopoietic site, splenic granulopoiesis could be more remarkable in some cases [34]. Furthermore, the importance of extramedullary granulopoiesis in LN has been emphasized in a recent study [10]. Extramedullary hematopoiesis and its control mechanism under the lupus circumstance are worth exploring in the future.

GSDMD is famous as a pore-forming protein which causes pyroptosis, while accumulating studies revealed its lysis-independent functions [15, 49]. Lupus-related damage-associated molecular patterns (DAMPs) may increase GSDMD’s expression through inflammasomes [50], yet there is no clear evidence linking it to increased pyroptosis in lupus [51], hinting at possible non-lytic roles of GSDMD in such conditions. It has been reported that low-level GSDMD-N can form small oligomers in plasma membrane which allow ion fluxes [52], drawing attention recently to how GSDMD-triggered calcium entry can initiate processes like membrane repair [53], coagulation cascade[54] and mucin secretion [55]. In addition to these events, we herein report a novel mechanism that GSDMD deficiency prompts granulocytic differentiation in myeloid progenitors through restraining calcium entry into cytoplasm. This process is partially reversed by low-dose ionomycin treatment, which serves as a classic activator of SOCE, facilitating Ca^2+^ release into the cytosol [56].Importantly, GSDMD-N interacts with phosphoinositides (PIPs) including phosphatidylinositol 4,5-bisphosphate [4, 5] and phosphatidylinositol 4-phosphate [57–59], which are pivotal in the SOCE by regulating the location of STIM1 to endoplasmic reticulum-plasma membrane junctions [60, 61]. These results support that GSDMD plays a key role in modulating intracellular calcium homeostasis by its pore-forming or lipid-binding functions. Calcium signaling has been reported to regulate cell differentiation positively or negatively in a variety of cell types. For example, intracellular calcium is essential for Treg cells differentiation into effector population and keratinocyte differentiation [35, 36], but limits the differentiation of B cells and adipocytes [37, 38]. As for myeloid progenitor cells, calcium and calcineurin signaling have been shown to inhibit cell cycle progression of GMPs to regulate myeloid cell differentiation [62]. We thus detected the cell cycle of NB4 cells and found that GSDMD knockdown increased proliferation and colony formation, indicating that GSDMD may regulate granulopoiesis in a cell cycle-dependent manner. Such notion was also supported by transcriptome sequencing data from lupus models, which showed myeloid GSDMD-deficiency drove an upregulation of mitotic cell division genes and downregulation of cytosolic calcium concentration genes. Interestingly, we observed Cebpe and Gfi1, two transcription factors crucial for early neutrophil differentiation [34], were up-regulated after GSDMD deficiency (data not shown), suggesting a potential intermediate mechanism of GSDMD-regulated granulopoiesis. Further studies are needed to make these clear.

GSDMD absence caused exacerbated autoimmunity in our study. The culprit is the altered heterogeneity of neutrophils derived from enhanced granulopoiesis. Granulopoiesis upon stress causes changes in the neutrophil subpopulations which affect disease progression. For instance, in infectious or tumor diseases, the growing immature neutrophils derived from emergency granulopoiesis are closely associated with poor prognostic outcomes [34, 63]. Although immature neutrophils are thought to lack some of the effector features of terminally differentiated cells [64], their functions may be altered under disease challenge. A recent study reported that neutrophils with immature signatures exhibited stronger effector functions including oxidative burst, NETosis, phagocytosis and pro-coagulation effects in aged poststroke models [65]. Besides, immature neutrophils isolated form tumor-bearing mice showed an increase ability for NETosis in challenge with A23187 compared to mature neutrophils [41]. These findings suggest that immature neutrophils under pathological conditions may possess capabilities that are not inferior and, in some cases, even stronger than those of mature neutrophils. Interestingly, our results proved that GSDMD deficiency drove an abnormal increase of immature neutrophils and an upregulation of NETs markers compared with wild-type littermates, in accordance with the first study addressing GSDMD role in SLE pathogenesis [13]. These findings prompt a dissection that targeting GSDMD may promote granulopoiesis which in turns to support inflammatory responses and autoimmunity in the context of LN.

There are some limitations in our research. Firstly, we performed the transcriptome sequencing using splenic myeloid cells to discover granulopoiesis after GSDMD deficiency. As bone marrow is an important site for hematopoiesis, the lack of medullary transcriptome sequencing may miss some information about GSDMD’s roles in medullary hematopoietic cells. Moreover, we explored the function of GSDMD in granulopoiesis of myeloid-specific deficient mice. A competitive reconstitution assay is needed to better describe the cell intrinsic effect of GSDMD, and myeloid progenitors-conditional deficient mice are of higher value to study lupus-related granulopoiesis in future studies. Besides, we did not explore the upstream mechanism of GSDMD. It was reported that inflammatory caspases (eg. caspase-1 and caspase-8) mediated the cleavage of GSDMD, which usually led to GSDMD-driven cell death [66, 67]. However, whether these molecules are involved in GSDMD-regulated granulopoiesis is unknown and worth exploring in the future, especially considering the important roles of inflammasomes in lupus progress [68]. Finally, we did not compare the effect of GSDMD ablation on granulopoiesis in different stages of LN course and the role of neutrophils in different maturities in the disease, which will be beneficial for precise intervention of GSDMD.

In conclusion, our study revealed that myeloid-intrinsic GSDMD deficiency could drive granulopoiesis by reducing calcium influx in myeloid progenitor cells, and promote neutrophil-mediated renal injury in lupus milieu (Fig. 6E). Our data provide a new perspective in understanding the non-lytic-cell-death function of GSDMD in SLE/LN pathogenesis, and establish a novel idea that targeting GSDMD should be carefully considered as it may trigger deregulated granulopoiesis in the development of SLE/LN.

## Conclusions

We found GSDMD is highly expressed in myeloid cell but exert a protective effect in the development of lupus nephritis. Importantly, the myeloid GSDMD deficiency leads to a marked increase of neutrophils output in spleen and bone marrow, which may be explained by the restricted intracellular calcium level which leads to aberrant self-renewal and a skewed granulocytic differentiation in myeloid progenitor cells. These insights shed light on the intricate and multifaceted role of GSDMD in modulating myeloid cell differentiation through ion fluxes, thereby offering novel perspectives on the role of GSDMD in the immunopathology of lupus.

### Supplementary Information

Supplementary Material 1.

## Data Availability

Data is provided within the manuscript or supplementary information files. The RNA sequencing data generated and/or analyzed during the current study are available from the corresponding author upon reasonable request.

## Abbreviations

GSDMD: Gasdermin D
SLE: Systemic lupus erythematosus
LN: Lupus nephritis
cGVH: Chronic graft-versus-host
NTS: Nephrotoxic serum
GMPs: Granulocyte/macrophage progenitors
ATRA: All-trans-retinoic acid
NETs: Neutrophil extracellular traps
NZB/W: New zealand black × white
GBM: Glomerular basement membrane
qRT-PCR: Quantitative reverse transcription PCR
DEG: Differentially expressed genes
CFU: Colony-forming unit
ACR: Albumin-to-creatinine ratio
PAS: Periodic acid-Schiff
BUN: Blood urea nitrogen
DC: Dendritic cells
pDCs: Plasmacytoid dendritic cells
RNA-seq: Whole-transcriptome sequencing
CMPs: Common myeloid progenitors
MPO: Myeloperoxidase
cfDNA: Cell-free DNA
MFI: Mean fluorescence intensity
CitH3: Citrullinated histone H3
SLEDAI: Systemic lupus erythematosus disease activity index
DAMPs: Damage-associated molecular patterns
PIPs: Phosphoinositides
