# Supplementary material for: Gasdermin D deficiency aborts myeloid calcium influx to drive granulopoiesis in lupus nephritis
Source: Cell Commun Signal. 2024 Jun 3;22:308. doi: 10.1186/s12964-024-01681-z (PMC11149269; doi:10.1186/s12964-024-01681-z)
Supplement: Supplementary file 1 — Supplementary Material 1. [file 12964_2024_1681_MOESM1_ESM.docx]

**Supplemental Materials**

**
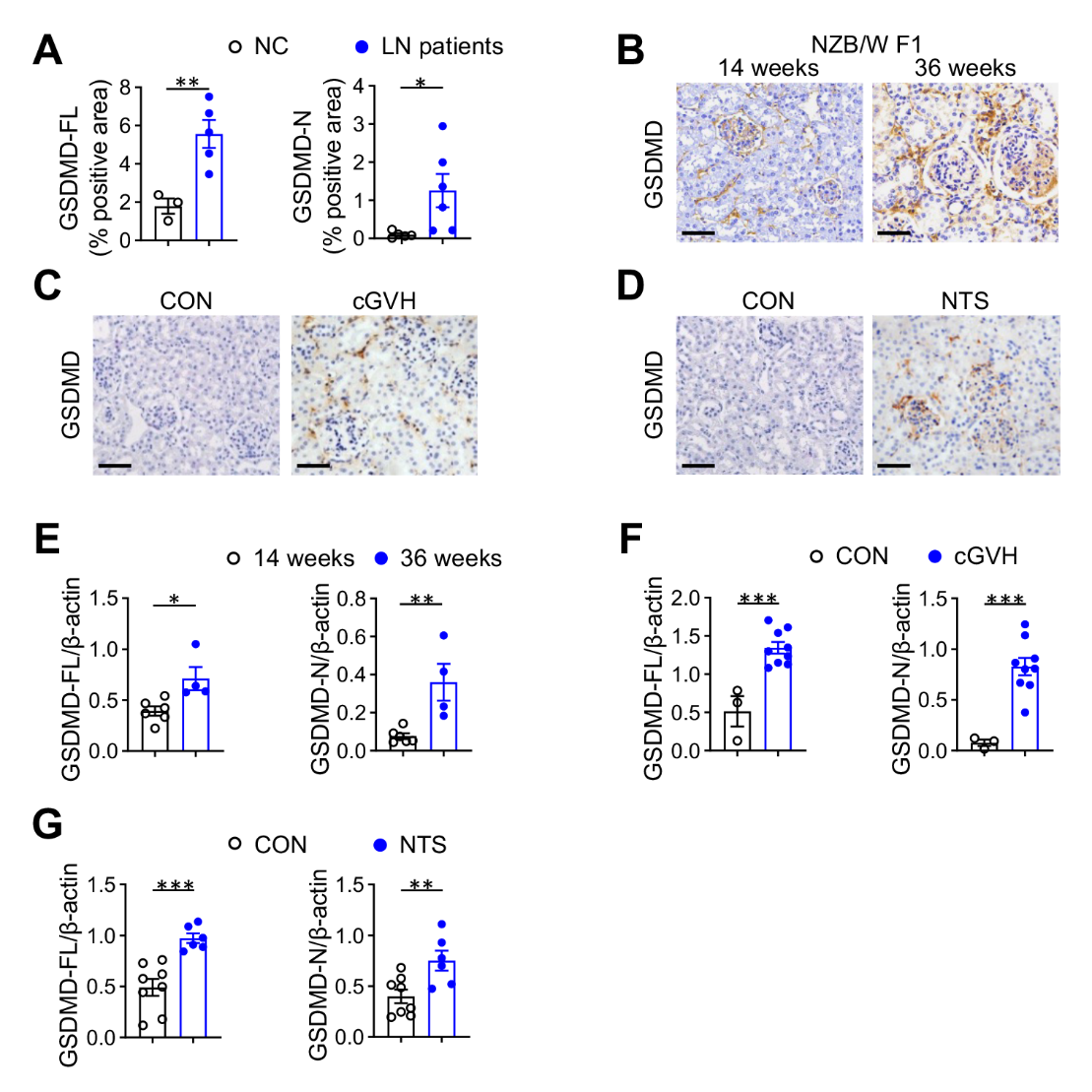
**

**Figure 1—Additional file 1. GSDMD expression is upregulated in human and mouse LN kidneys.** (A) Statistical graphs of immunoblotting of GSDMD-FL and GSDMD-N in renal protein extracts of LN patients and their normal control (NC) (n = 3-6 per group). (B-D) Immunochemical representative images of GSDMD pattern in renal section from NZB/W F1 mice at 14 weeks and 36 weeks (B), cGVH and control (CON) mice (C), or NTS and CON mice (D). Scale bars: 50 μm. (E-G) Statistical graphs of immunoblotting of GSDMD-FL and GSDMD-N in renal protein extracts of NZB/W F1 mice at 14 and 36 weeks (E) cGVH and CON mice (F), or NTS and CON mice (G). (n = 3 or 4 per group). Data are shown as mean ± SEM. Student’s t tests was used for statistical analysis. ****P* < 0.001; ***P* < 0.01; **P* < 0.05.


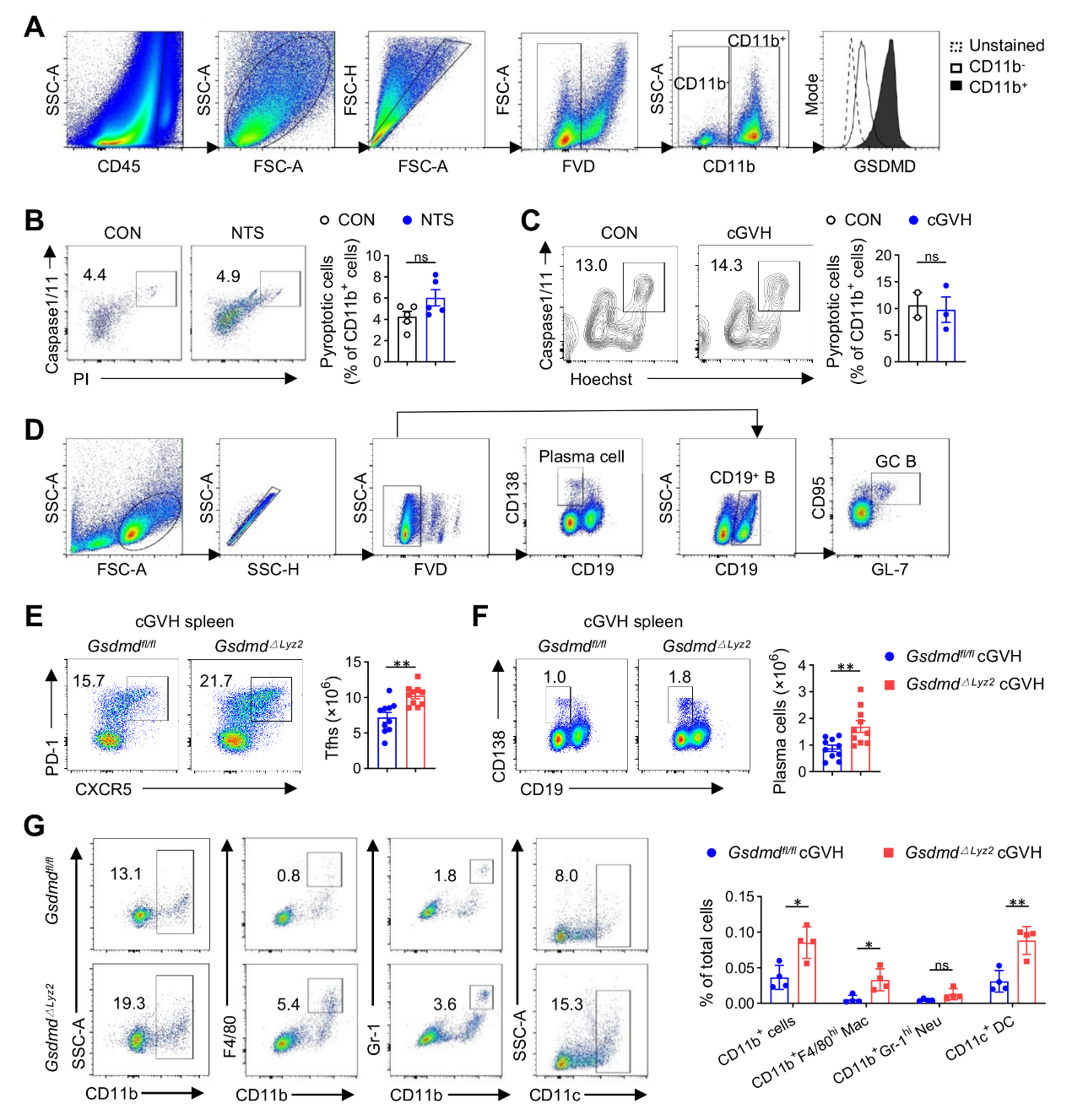


**Figure 2—Additional file 1. Myeloid GSDMD deletion aggravates renal immune infiltration and systemic autoimmunity.** (A) Gating strategy to identify myeloid cells and GSDMD expression. (B) Flow analysis of pyroptosis cells (Caspase1/11^+^PI^+^) in kidneys of control (CON) and NTS mice (n = 5 per group). (C) Flow analysis of pyroptosis cells (Caspase1/11^+^Hoechst^+^) in kidney of CON and cGVH mice (n = 2 or 3 per group). (D) Gating strategy to identify B cells. (E-F) Representative image of flow cytometric analysis with the counts of CD4^+^CXCR5^+^PD-1^+^ Tfhs (E) and CD19^-^CD138^+^ plasma cells (F) from the spleens of *Gsdmd^fl/fl^* and *Gsdmd^△Lyz2^* cGVH models (n = 10 per group). (G) Representative flow cytometric plots and quantitative analysis of the percentages of CD11b^+^ cells, CD11b^+^F4/80^hi^ macrophages (Mac), CD11b^+^Gr-1^+^ neutrophils (Neu), and CD11c^+^ dendritic cells (DC) among total cells in the kidneys of *Gsdmd^fl/fl^* and *Gsdmd^△Lyz2^* NTS mice (n = 2 or 3 per group). Data are shown as mean ± SEM. Student’s t test was used for statistical analysis. ***P* < 0.01; **P* < 0.05; ns, not significant.

**
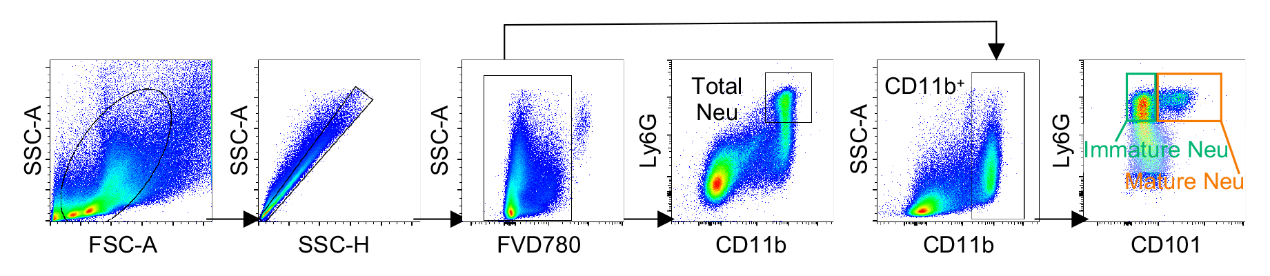
**

**Figure 3—Additional file 1. Flow gating strategy for neutrophils.** Gating strategy to identify total neutrophils, immature neutrophils and mature neutrophils.


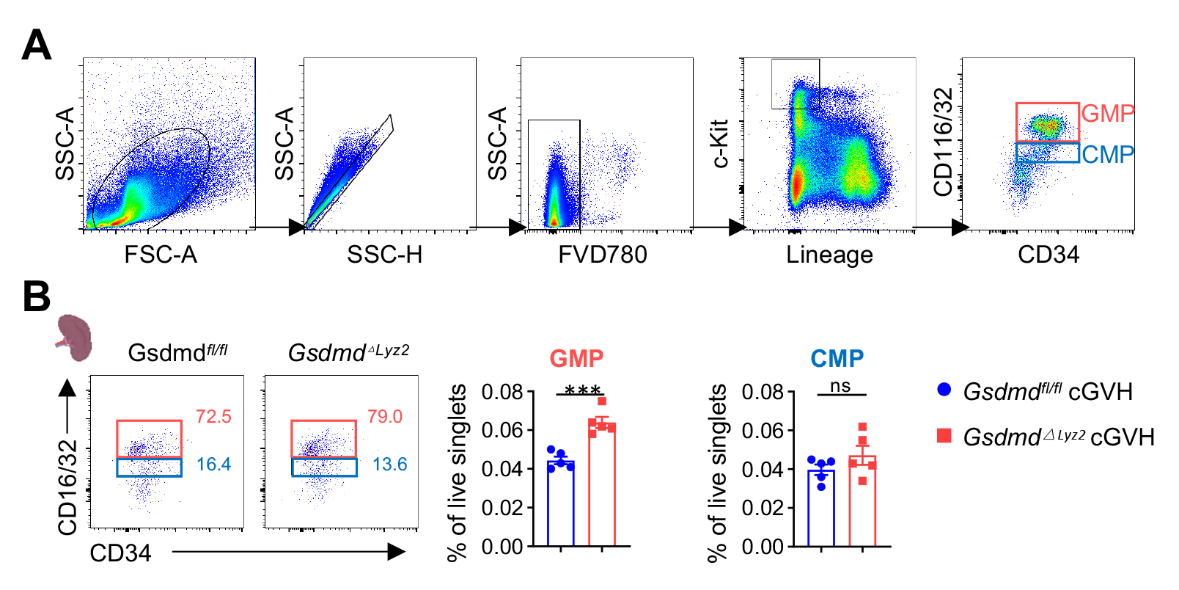


**Figure 4—Additional file 1. Myeloid GSDMD deficiency leads to an increase of precursor cells in cGVH model.** (A) Gating strategy to identify GMPs and CMPs. (B) Flow cytometric analysis of GMPs and CMPs in the spleens of cGVH model induced in *Gsdmd^fl/fl^* and *Gsdmd^△Lyz2^* mice (*n* = 5 per group). Data are shown as mean ± SEM. Student’s t tests was used for statistical analysis. ****P* < 0.001; ns, not significant.

**Table 1: Baseline characteristics of LN patients enrolled** **for immunochemical staining.**

| Characteristic | LN patients (n=6) |
| --- | --- |
| Age, years, median (range) | 27 (16-34) |
| Female, n (%) | 6 (100%) |
| Male, n (%) | 0 |
| Duration of SLE, months, median (range) | 9.5 (0.3-72) |
| Histopathological classification*, n (%) |  |
| II | 0 |
| III | 0 |
| IV-G | 6 (100%) |
| IV+V | 0 |
| V+III | 0 |
| V | 0 |
| SLEDAI, median (range) | 17.5 (6.0-22.0) |
| Proteinuria, g/day, median (range) | 3.89 (0.88, 12.92) |
| SCr, μmol/L, median (range) | 198.5 (96.0, 462.0) |
| Anti-dsDNA, IU/ml, median (range) | 138.5 (2.1, 476.1) |
| C3, g/L, median (range) | 0.36 (0.27, 0.46) |
| C4, g/L, median (range) | 0.13 (0.04, 0.27) |
| Medications, n (%) |  |
| Glucocorticoid | 6 (100%) |
| Hydroxychloroquine | 2 (33%) |
| Belimumab | 0 |
| Cyclophosphamide | 0 |
| Mycophenolate | 0 |
| Tacrolimus | 0 |
| Cyclosporine | 0 |
| Azathioprine | 0 |

* ISN/RPS classification of lupus nephritis (LN) in 2003.

Data are median (range) or n (%).

SCr, serum creatinine; SLEDAI, Systemic Lupus Erythematosus Disease Activity Index 2000.

**Table 2: Baseline characteristics of LN patients enrolled for GSDMD detecton in blood neutrophils.**

| Characteristic | LN patients (n=34) |
| --- | --- |
| Age, years, median (range) | 29.5 (16-70) |
| Female, n (%) | 26 (76%) |
| Male, n (%) | 8 (24%) |
| Duration of SLE, months, median (range) | 30.0 (0.25-192) |
| Histopathological classification*, n (%) |  |
| II | 1 (3%) |
| III | 1 (3%) |
| IV-G | 19 (56%) |
| IV+V | 5 (15%) |
| V+III | 3 (9%) |
| V | 5 (15%) |
| SLEDAI, median (range) | 10 (2-24) |
| Proteinuria, g/day, median (range) | 1.52 (0.06, 10.31) |
| SCr, μmol/L, median (range) | 90.0 (38.0, 615.0) |
| Anti-dsDNA, IU/ml, median (range) | 48.0 (2.0, 300.0) |
| C3, g/L, median (range) | 0.58 (0.14, 1.28) |
| C4, g/L, median (range) | 0.14 (0.02, 0.35) |
| Medications, n (%) |  |
| Glucocorticoid | 34 (100%) |
| Hydroxychloroquine | 25 (74%) |
| Belimumab | 10 (29%) |
| Cyclophosphamide | 9 (27%) |
| Mycophenolate | 7 (21%) |
| Tacrolimus | 5 (15%) |
| Cyclosporine | 1(3%) |
| Azathioprine | 1(3%) |

* ISN/RPS classification of lupus nephritis (LN) in 2003.

Data are median (range) or n (%).

SCr, serum creatinine; SLEDAI, Systemic Lupus Erythematosus Disease Activity Index 2000.
